# The monoicous secondarily aquatic liverwort *Ricciocarpos natans* as a model within the radiation of derived Marchantiopsida

**DOI:** 10.3389/fpls.2023.1260596

**Published:** 2023-11-28

**Authors:** Shilpi Singh, John L. Bowman

**Affiliations:** ^1^ School of Biological Sciences, Monash University, Melbourne, VIC, Australia; ^2^ Australian Research Council (ARC) Centre of Excellence for Plant Success in Nature and Agriculture, Monash University, Melbourne, VIC, Australia

**Keywords:** Ricciocarpos natans, liverwort, aquatic macrophytes, abscisic acid, evo devo

## Abstract

Liverworts represent one of six embryophyte lineages that have a Devonian, or earlier, origin, and are, at present, represented by only *Marchantia polymorpha* as an established model. *Ricciocarpos natans* is a secondarily monoicous aquatic liverwort with a worldwide distribution, being found on all continents except Antarctica. *Ricciocarpos*, a monotypic genus, forms a sister relationship with *Riccia*, the largest genus of the Marchantiopsida (~250 species), diverging from their common ancestor in the mid-Cretaceous. *R. natans* is typically found on small stagnant ponds and billabongs (seasonal pools), where it assumes a typical ‘aquatic’ form with long scale keels for stabilization on the water surface. But, as water bodies dry, plants may become stranded and subsequently shift their development to assume a ‘terrestrial’ form with rhizoids anchoring the plants to the substrate. We developed *R. natans* as a model to address a specific biological question — what are the genomic consequences when monoicy evolves from ancestral dioicy where sex is chromosomally determined? However, *R. natans* possesses other attributes that makes it a model to investigate a variety of biological processes. For example, it provides a foundation to explore the evolution of sexual systems within *Riccia*, where it appears monoicy may have evolved many times independently. Furthermore, the worldwide distribution of *R. natans* postdates plate tectonic driven continent separation, and thus, provides an intriguing model for population genomics. Finally, the transition from an aquatic growth form to a terrestrial growth form is mediated by the phytohormone abscisic acid, and represents convergent evolution with a number of other aquatic embryophytes, a concept we explore further here.

## Introduction

Why does one choose a particular species as a model system? For most of the 20^th^ century plant biologists chose species based on attributes that facilitate experimental approaches to answer a specific biological question (Sussex, 1998). This approach led to a plethora of different species being investigated, each suited the questions being investigated. At one point, it became so parochial that each researcher worked on a different species, and as Ian Sussex once related, he was thinking about working on a particular species of plant, but was told by others that he should not, as that was professor X’s species. Despite the parochialism, during that time a few species, namely maize, petunia and snapdragon, gained some traction as models, especially among geneticists (Stubbe, 1966; Rhoades, 1984; Coe, 2001; Schwarz-Sommer et al., 2003; Candela and Hake, 2008; Vandenbussche et al., 2016). It was not until the 1980’s that the plant science community converged upon a dominant model system, *Arabidopsis thaliana* (Meyerowitz and Pruitt, 1985; Page and Grossniklaus, 2002; Somerville and Koornneef, 2002; Provart et al., 2015; Prunet and Meyerowitz, 2016). Subsequently, research with a small number of species amenable to genetic and molecular approaches facilitated rapid advances in our understanding of plant development, physiology, and even ecology. However, this canalization also led to other aspects of plant biology for which the model species were not appropriate, such as mycorrhizal fungal interactions and broader questions in evolution and ecology being neglected. With the advances of genomic sequencing and development of genome editing in the past decade, we are now in a position to return to the broader plant biology perspective of last century, where a wide spectrum of species could be developed as models given the specific biological question at hand. It is in this vein that we began research on the liverwort *Ricciocarpos natans*.

## Materials and methods

### Ricciocarpos culture

To determine optimal growth conditions in the laboratory (i.e. to mimic morphologies observed in nature), *Ricciocarpos* was growth in axenic aquatic culture, with liquid media containing various concentrations of Gamborg B-5 basal medium [PhytoTech Labs; www.phytotechlab.com; (Gamborg et al., 1968)], pH 6.0, with concentrations of 1x, 1/2, 1/4, 1/6, 1/8, 1/10, and 1/12 tested. Growth was under a 16-hour photoperiod at 20˚C. Light intensity was varied by growing plants at different distances from the light source. Conditions of 1/8 B-5 media and a light intensity of 80 µmol. m^-2^s^-1^ resulted a typical aquatic morphology with purple scale production. Addition of ABA at a concentration of 0.1µM to the above media successfully induced terrestrial characteristics of *Ricciocarpos* growing in liquid media; for the differential gene experiment, the addition of ABA was a single event at the initiation of the growth period.

### Ricciocarpos accession relationships

The phylogenetic tree of *Ricciocarpos* was constructed using nucleotide sequences of 6 genes (nuclear: LOX1; chloroplast: *rbcL*, *rps4*, cpITS, *trn*L-F, 26S); while the Jerrybomberra Creek and Butner NC accessions were represented by most genes, the other accessions were represented by as few as one gene. Each sequence alignment was manually trimmed to exclude ambiguously aligned regions. The best substitution models for each of the six alignments obtained using the “optimize” function in raxmlGui2.0 (Edler et al., 2020). The six alignments were concatemerized producing a matrix consisting of 12,465 nucleotides representing 11 species/isolates. A maximum likelihood phylogeny was constructed, with nodal support calculated after 1000 replications, using raxmlGui2.0 (Edler et al., 2020).

### Annotation of Ricciocarpos genome

*Repeat Annotation*: RepeatModeler (Smit and Hubley, 2008-2015) (version 1.0.8_RM4.0.7) was used for *de novo* repeat family identification. The output was used as a repeat library for RepeatMasker version 4.0.9 (Smit et al., 2013-2015).

*RNA Extraction and Sequencing:* RNA for sequencing was extracted from *R. natans* by submerging whole plants in liquid nitrogen and using a mortar and pestle to grind the tissue into a powder. For each line, 100mg of tissue was processed with the RNeasy mini kit (Qiagen), as per the manufacturer’s instructions for purification of total RNA from plant tissues. The total RNA for each sample was quantified with the NanoDrop 2000 (Thermo Scientific). Library preparation used polyA mRNA selection and MGIEasy stranded mRNA chemistry. Sequencing used MGI Tech MGISEQ-2000RS hardware (400 million raw reads per lane, 100-pb paired-end reads).

*Gene Prediction*: A total of 106,736 transcript assemblies were made from ∼57M pairs of paired-end Illumina RNA-seq reads with Trinity software-v2.12.0 described in Chapter 3. Ab-initio gene predictions were generated by AUGUSTUS-3.3.3 (Stanke et al., 2008). 37,626 transcript assemblies were constructed with RNA-seq-assisted prediction by PASA software (Haas et al., 2003) using RNA-seq transcript assemblies. Homology based gene prediction was done with EXONERATE alignments with the Marchantia v6.1 protein dataset to a repeat-soft-masked *Ricciocarpos* genome using RepeatMasker (Smit et al., 2013-2015). The Repeat library was generated using RepeatModeler (Smit and Hubley, 2008-2015).

The EVidenceModeler software, which combines ab-initio gene predictions, protein and transcript alignments into weighted consensus gene structures, was used to obtain consensus gene structures (Haas et al., 2008) in *R. natans*. Resultant gene structure annotations were updated by PASA. As *Marchantia* is used as model liverwort for comparison with *R. natans*, and is functionally well annotated, PASA-improved gene model proteins were subject to protein homology analysis to *Marchantia* to retrieve functional annotation of genes.

### Differential gene expression analysis

RNA-seq filtered libraries, in triplicate, were used for each sample. Scaffold level assembly of *Ricciocarpos* (https://genomevolution.org/coge/; Genome id65508) was used as the reference for mapping filtered transcripts to *R. natans* genome assembly using samtools (Li and Durbin, 2009). Transcript abundance was estimated using HT-Seq count with -no strand specific parameter (-s = no) and other parameters were kept as default. Differential gene expression (DGE) analysis of ABA treated plants (terrestrial) in contrast to no ABA (aquatic) was performed with DESeq2 (Love et al., 2014).

## *Ricciocarpos natans*, an aquatic monoicous liverwort

**Figure 1:**
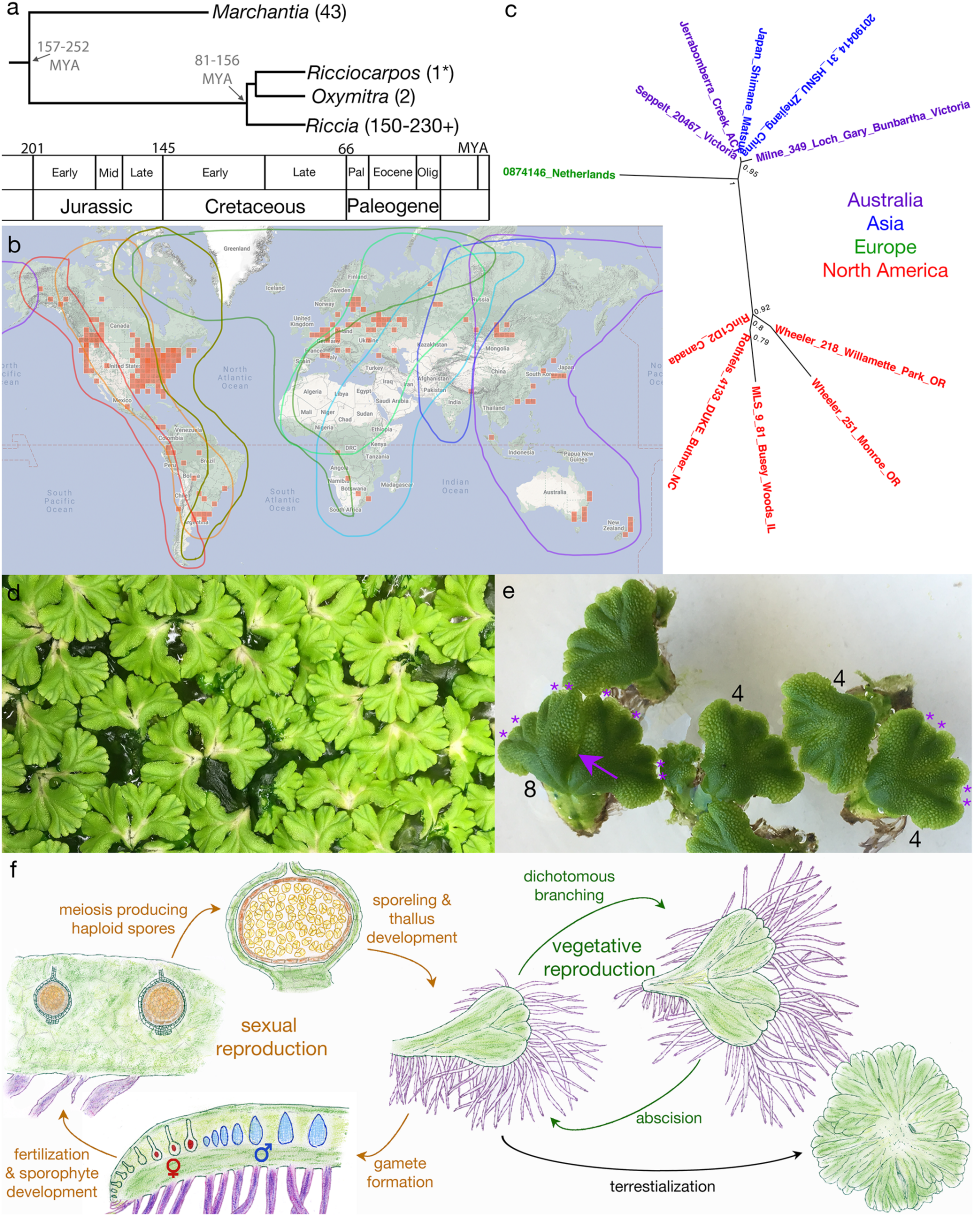
Overview of *Ricciocarpos*. **(A)** Phylogenetic relationship of *Ricciocarpos* to its closest extant relatives and to *Marchantia*, with approximate divergence times indicated (MYA, million years ago). Tree topology and estimated times are based on an analysis of 12 genes of mixed organellar and nuclear origin and calibrated with the known fossil record, with 95% posterior density intervals indicated [from (Villarreal et al., 2016)]. Numbers in parentheses indicate number of species in genera. **(B)** Present day distribution (red squares) of *Ricciocarpos* displayed at inaturalist.org (19 August 2022). The eight major flyways for migrating birds are outlined in different colours (Boere and Stroud, 2006). **(C)** Relationship between accessions of *R. natans*: the genome of the present paper is from Jerrabombera Creek, ACT (Australia), while the 1kp transcriptome (Wickett et al., 2014) was from plants isolated near Butner, NC (USA), and other accessions are from data in Genbank. Names are colour-coded based on geographical location. **(D)** Clonal population of *Ricciocarpos* growing in water amongst algae. **(E)** Dichotomous branching and separation of thallus fragments. The numbers indicate the number of apical meristems in the thalli, with the asterisks demarking them in two thalli. the arrow indicated where this thallus with eight apices will separate. **(F)** The life cycle of *Ricciocarpos*; sexual reproduction in brown and vegetative reproduction in green. Sporophyte tissues are shown in brown shades, whereas gametophyte tissues are green, except scales (purple), egg cells (red), antheridia (blue). Plants most often undergo vegetative reproduction *via* successive cycles of dichotomous branching and subsequent abscision of plants along the midline. If plants become stranded on a terrestrial substrate, production of long scales ceases and rhizoid are produced; in addition, abscision following branching no longer occurs, with plants forming a rosette. Growth in inductive conditions (aquatic growth and likely longer warmer days in spring) leads to production of first antheridia and then archegonia along the dorsal midline. Self- or cross-fertilization leads to production of sporophytes, again along the dorsal midline and essentially enclosed by the maternal gametophyte. As sporophytes lack both foot and seta, maternal nutrient contributions must be transferred *via* the calyptra. Following meiosis and release of spores from tetrads, the unistratose capsule wall (dark brown) breaks down, releasing the spores into a cavity in the maternal gametophyte to be dispersed as the gametophyte senesces. Adapted from (Bischoff, 1835; Garber, 1904).

Liverworts are one of three bryophyte lineages (liverworts, mosses, hornworts) and comprise one of six land plant lineages that diverged from one another in the Devonian or earlier (Bowman, 2022). *Ricciocarpos natans*, hereafter *Ricciocarpos*, is a monoicous, largely aquatic, complex thalloid liverwort (Marchantiopsida) with a cosmopolitan distribution (**Figures 1A–C**). The genus is monotypic, being comprised of a single described species. Due to its resemblance to species of the genus *Riccia*, *Ricciocarpos* has traditionally been placed as one of the two genera of the Ricciaceae (Schuster, 1992), however, phylogenetic analyses using sequence data suggest it is more closely related to another genus, *Oxymitra*, and that they together are sister to *Riccia* [**Figure 1A**, (Villarreal et al., 2016)]. As liverworts were ancestrally dioicous and terrestrial, both monoicy and its aquatic habit are derived characters. However, development of *Ricciocarpos* as a model system could serve as a model for species in the genus *Riccia*, the largest genus by species number within the Marchantiopsida.

The morphology, anatomy and development of *Ricciocarpos* is similar to that of other complex thalloid liverworts. Growth occurs from a single apical cell in the shoot meristem, with the thallus undergoing periodic dichotomous branching [**Figures 1D, E** (Leitgeb, 1879; Garber, 1904)]. The dorsal surface is occupied by air chambers separated by unistratose (single cell layer) walls and forming an elaborate aerenchyma, with those at the dorsal surface having a complex air pore (Leitgeb, 1879; Kronestedt, 1981; Kronestedt, 1982a; Kronestedt, 1982b). The air chambers form schizogenously, i.e. *via* localized cell separation (Barnes and Land, 1907; Hirsh, 1910). The air in these chambers provides buoyancy such that the plants float on the water surface. As is typical of the Marchantiopsida, oil body cells are found as idioblasts (isolated cells differing from their neighbours) containing a single oil body (Kronestedt, 1983), and these likely function to deter herbivory as has been described for *Marchantia* (Kanazawa et al., 2020; Romani et al., 2020).

**Figure 2:**
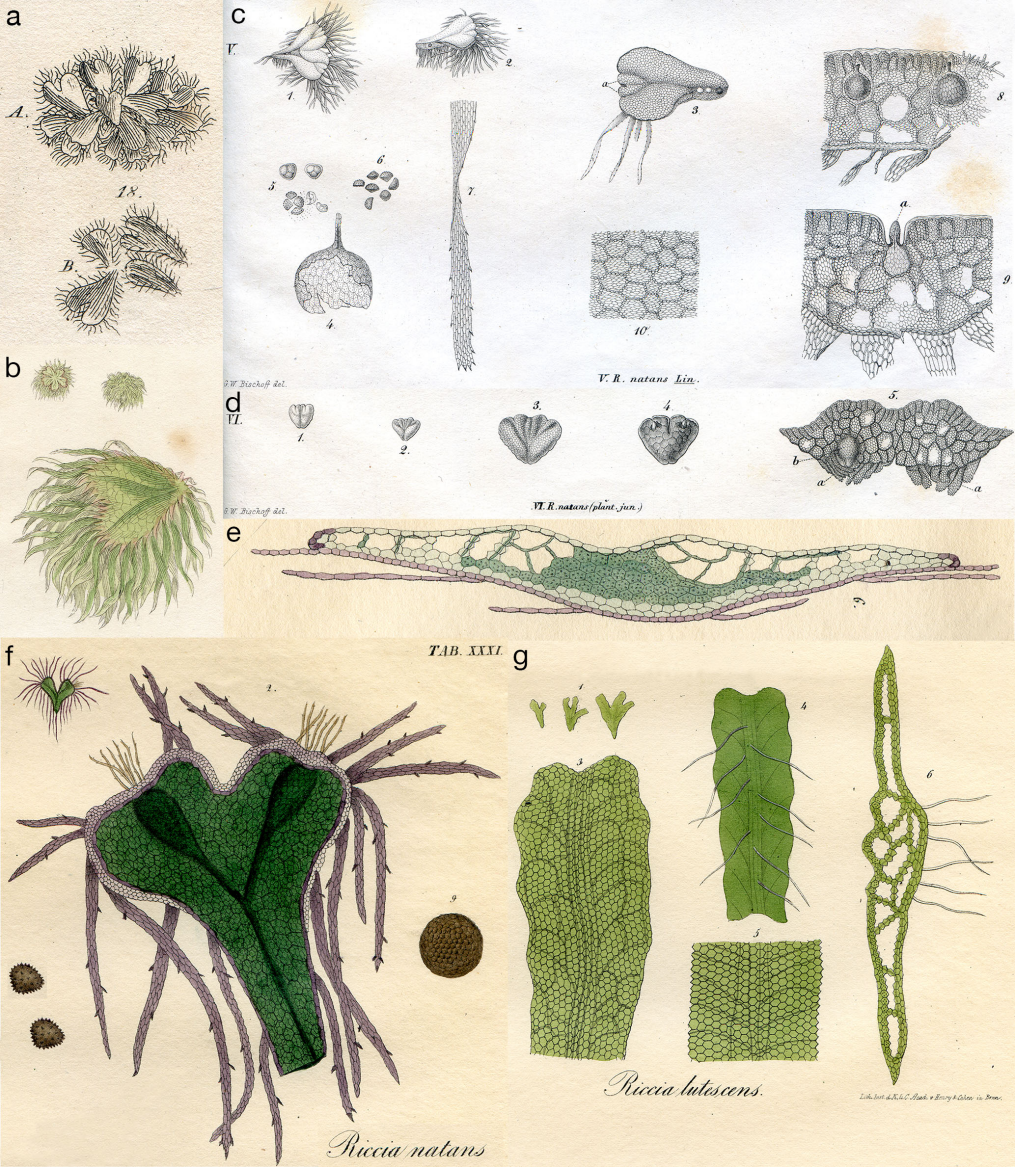
**(A)** A. dorsal surface; B. ventral surface; Plate 78, fig. 18 from Dillenius (Dillenius, 1741). **(B)** *Riccia natans* (Fringed *Riccia*), plate 77 in Smith’s English Botany (Smith, 1804). **(C)** *Riccia natans*, Plate LXXI, fig. V of (Bischoff, 1835). (1) Mature frond; (2-3) fronds split into two halves along the intermediate groove, each half consisting of two unequal parts; (4) the sporangium; (5) developing spores; (6) mature spores; (7, 10) scale; (8) sporangium within thallus; (9) thallus cross section within antheridium **(A)**. **(D)** *Riccia natans* (plant juvenile), Plate LXX, fig. VI of (Bischoff, 1835). (1-3) Young fronds with quadricrenate apex, convex on both sides, attenuated on the outer edge, with an intermediate groove running out into two lateral grooves from the notches; (4) ventral side of 3; (5) frond cross section with young scales **(A)**. **(E, F)** *Riccia natans*, from Plate XXXI of (Lindenberg, 1836). **(E)** 6. cross section of mature frond. **(F)** 2. Mature frond; (9) sporangium; (left) mature spores. **(G)** *Riccia lutescens*, from Plate XXVI of (Lindenberg, 1836). (1, 3) Mature frond; (4) ventral view; (5) enlarged view of 4; (6) thallus cross section.

**Figure 3:**
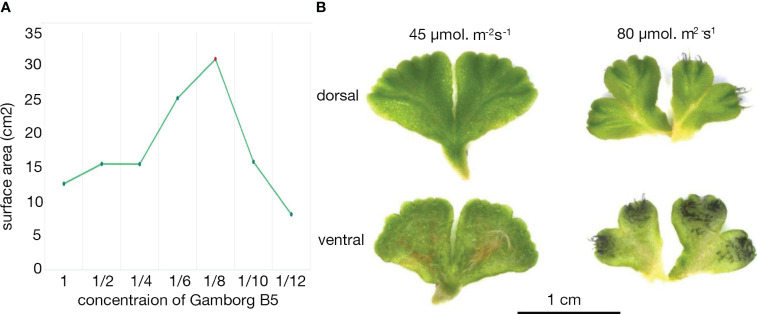
Growth of *Ricciocarpos* in culture. **(A)** Growth curve with varying Gamborg B-5 concentration. **(B)** Variation in induction of purple scales in *Ricciocarpos* with light intensity. The plate with ⅛ Gamborg B-5 media and higher light intensity produced growth with prominent purple scales compared to the plate with the same nutrient concentration and less intense light.

When *Ricciocarpos* is growing on an aquatic medium, ventral rhizoid production is suppressed, and large sword-like scales are produced that act as keels to stabilise the thallus on the water surface and prevent their overturning during windy periods [(Bischoff, 1835; Lindenberg, 1836; Kronestedt, 1981); e.g. **Figure 2**, **Figures 3A, B**]. The scales are unistratose, several cells wide (0.2-0.6 mm), and often with their length (10 mm) exceeding the width of the thallus (Kronestedt, 1981). The scales are often deeply pigmented, with a reddish-purple pigment that can almost appear black (Dillenius, 1741; Lindenberg, 1836; Schmidel, 1793). The pigment has been named riccionidin and its biosynthesis is related to that of the anthocyanin pathway (Kunz et al., 1994; Kunz and Becker, 1995; Albert et al., 2018; Kubo et al., 2018), and recent work on *Marchantia* has shown it to be an auronidin (Berland et al., 2019). This pigment appears to be polymerized in the cell wall, and thus provides both a possible sunscreen and also contributes mechanically to the stiffness of the scales (Kunz et al., 1994; Berland et al., 2019). Another attribute of the aquatic form is the periodic separation of thalli (Bischoff, 1835; Garber, 1904; Lewis, 1906; Pickett, 1925; Schmidel, 1793), *via* abscision and presumably involves programmed cell death (**Figures 1D–F**). Typical thalli have four shoot apices, and when each of these apices branch a short-lived thallus with eight shoot apices is formed that then undergoes abscission to produce two thalli with four apices once again [**Figures 1D–F**, (Ferreyra, 1990)]. This mode of vegetative reproduction allows for rapid proliferation of thalli on the water surface, with each ‘individual’ being able to float independently of the others. Consistent with its largely aquatic ecology, *Ricciocarpos* has lost the ability to form mycorrhizal fungal interactions (Stahl, 1949; Ligrone et al., 2007).

When *Ricciocarpos* is growing on a terrestrial medium, scale production is suppressed and instead unicellular thin-walled rhizoids as long as 15 mm are produced and these anchor the plant to the substrate (Kronestedt, 1981). In addition, the terrestrial form does not undergo fragmentation *via* abscission, but rather stay together forming a tight circular rosette, similar to many *Riccia* species (Bischoff, 1835; Garber, 1904; Lewis, 1906; Pickett, 1925).

*Ricciocarpos* is monoicous, producing first antheridia and then archegonia along the dorsal furrow [**Figure 1F**; (Garber, 1904)]. As the sex organs are produced in a temporally distinct manner, there is some scope for outcrossing. The heterochronic production of sex organs is likely what led to some early researchers to suggest dioicy (Campbell, 1895; Leitgeb, 1879). It is this derived feature, the evolution of monoicy from ancestral dioicy, without a major karyotype change [n = 9 (Siler, 1934), from the ancestral state of liverworts (Berrie, 1960)], that induced us to develop *Ricciocarpos* as a model to investigate the genomic consequences of a shift in sexual systems (Singh et al., 2023). The antheridia and archegonia are produced along the dorsal furrow in a single row (Leitgeb, 1879; Garber, 1904; Lewis, 1906; Rieth, 1959), and their development are typical of the Marchantiopsida (Leitgeb, 1879; Garber, 1904; Lewis, 1906). Following fertilization the sporophyte develops essentially embedded within the maternal thallus [**Figure 1F**; (Garber, 1904; Lewis, 1906; Rieth, 1959)]. The sporophytes consist of a capsule whose wall is unistratose, and largely lack both a foot and seta (**Figure 1F**). All spore mother cells under meiosis producing haploid spores [about 500 per sporangium; (Rieth, 1959)], with no evidence of elaters (**Figure 1F**). Following spore formation, the capsule wall breaks down releasing the spores into a cavity in maternal gametophyte, with dispersal either due to degeneration of the maternal gametophyte or *via* bird consumption (see below).

## The discovery and early description of *Ricciocarpos*

The discovery and description of *Ricciocarpos* was given by Buddle in 1699 under the name ‘*Lichen parvus vernus cordiformis, ima parte fimbriatus, Lentis palustris modo aquae innatans*’ in his *Hortus Siccus*, a herbarium that he assembled. A description was first published by Jacob Petiver (Petiver, 1695-1703), “*Lens palustris Roris Solis foliis cordatis*, observed by my Reverend friend Mr. Adam Buddle in some ponds about Henley in Suffolk”, later corrected to ‘Hadley’ in Suffolk (Dillenius, 1741). It was later published under Buddle’s original description in John Ray’s *Synopsis methodica stirpium Britannicarum*, 3^rd^ edition, and who noted that “It was suspected that it is being eaten by insects or ducks” (Ray, 1724). The first published image (**Figure 2A**) was in Dillenius’s *Historia Muscorum* (Dillenius, 1741). With respect to the cryptogams, Linneaus was a great lumper and placed *Ricciocarpos* with *Riccia* under the name *Riccia natans* (Linné, 1770); the species epithet ‘natans’ is derived from the Latin word for ‘swimming’. As is often the case, Schmidel’s description (of what he called *Riccia capillata*) added much detail, where he noted the dorsal air chambers and the serrated and coloured (black) nature of the ventral scales, and further suggested that it multiplied *via* dissociation along ‘nerves’ separating the lobes (Schmidel, 1793). A much less detailed drawing is presented in Smith’s English Botany [**Figure 2B**; (Smith, 1804)]. Hooker was the first to describe the position of the sporophyte (Hooker, 1830), with Bischoff subsequently describing the structures of the sunken antheridia and sporophytes in some detail (**Figure 2C**), and also detailing the vegetative propagation *via* repeated division along the central channel of older plants into two plants (Bischoff, 1835). As with the case of many liverworts, new techniques for fixation and sectioning allowed Leitgeb to provide a detailed anatomical description of *Ricciocarpos*, with foci on development from the apical cell and the formation of air chambers (Leitgeb, 1879). Anatomical details of antheridia, archegonia and sporophyte development were clarified some years later (Garber, 1904; Lewis, 1906).

The genus name *Ricciocarpos* was coined by Corda (Corda, 1828) to separate *Ricciocarpos* from other *Riccia* species, but the first illustrations under the moniker *Ricciocarpos* are very poor [see Tab. 32. in (Sturm, 1832)]. Furthermore, Corda’s distinguishing characters were shown not to be diagnostic (Bischoff, 1835), and thus it was subsequently often placed back into the genus *Riccia*. However, likely due to its similarity with other water plants and a lack of attention to detail, *Ricciocarpos* was described under other genera, e.g. *Salviniella* (*natans*) (Hübener, 1834). C. S. Rafinesque described *Ricciocarpos* growing on ponds on Long island, NY as *Lemna dimidiata*, perhaps ironically as he was didactic in correcting other botanist’s nomenclature (Rafinesque, 1817). However, to his credit, Rafinesque also formulated a prescient early view of evolution: “*The truth is that Species and perhaps Genera also, are forming in organized beings by gradual deviations of shapes, forms and organs, taking place in the lapse of time. There is a tendency to deviations and mutations through plants and animals by gradual steps at remote irregular periods. This is a part of the great universal law of perpetual mutability in everything.*” (Rafinesque, 1833), which was later acknowledged by Darwin. Bischoff disparaged this proliferation of genus names, saying of some of his contemporaries, “*they could not resist the addiction to see their names behind a synonym, even if they were born as an invalid*”, and in his description of *Ricciocarpos* he wished “*to protect us and our descendants from Babylonian confusion*” (Bischoff, 1835). Furthermore, “*In order to finally prevent such a polyonomatomania, which might threaten to infiltrate our Riccia even further*”, Bischoff also drew “*attention to a plant which was discovered in February in the ditch of Lille near the Pont-de-France by Gay and (in the year 1834) to Prof. Al. Braun was notified*” (**Figure 2D**). Bischoff interpreted “*this plant, which at first glance could be taken to be a separate species, is in all probability only the Riccia natans in its youngest condition*” (Bischoff, 1835). Bischoff noted that while the dorsal surfaces were nearly identical, the ventral surfaces were different, which we now interpret to be due to the differences in the production of rhizoids rather than scales. The terrestrial form was actually described some years earlier as a distinct species, *Riccia lutescens*, that was “*found in abundance in an exsiccated swamp on the ground*” in western North Carolina (Schweinitz, 1821), consistent with the habitat of terrestrial *Ricciocarpos*. Bischoff’s observations appear the first to equate the aquatic and terrestrial forms of *Ricciocarpos* to the same species. Lindenberg, who produced stunning drawings of *Ricciocarpos* (**Figures 2E-G**), also stated that when floating in the water large purple scales were produced, but “if the plant floats completely and consistently on the water, it is absolutely rootless, like *R. fluitans* in the same case; but as soon as it approaches the bank, or rests on the mud, it drives thin, delicate, rounded, hair-shaped, often articulated root fibers” [**Figure 2E**, (Lindenberg, 1836)]. Remarkably, despite these observations, Lindenberg also listed *R. lutescens* as a distinct species, with no cross reference to *Ricciocarpos* [**Figure 2G**, (Lindenberg, 1836)]. While some equated the two forms as a single species (Lindberg, 1882), *R. lutescens* was often listed as a separate species in other publications into the mid-20^th^ century, despite Lewis conclusively demonstrating that the terrestrial form could be converted into the aquatic form (Lewis, 1906). An additional distinct species name for the terrestrial form, *Riccia velutina*, was also proposed in the mid-19^th^ century (Hooker, 1840). The original *R. lutescens* specimen of Schweinitz was typified as an isolectotype of *Ricciocarpos natans* (Stotler and Crandall-Stotler, 2017). A full account of the historical nomenclature of *Ricciocarpos*, including its orthographical variant (*Ricciocarpus natans*), has been previously described (Duthie and Garside, 1936; Perold, 1995).

## Distribution and ecology of *Ricciocarpos*

*Ricciocarpos* has a nearly cosmopolitan distribution, being found throughout temperate habitats on six continents in both hemispheres, absent from extreme alpine habitats, the Arctic and Antarctica [**Figure 1B**; (Scott, 1985; Schuster, 1992)]. While not widely reported from the tropics, it can be found in both the neotropics of the Americas and tropics of the old world in Africa (Jones, 1957; Bischler-Causse et al., 2005). Notably, not long after *Ricciocarpos* was being described across western Europe, e.g. Germany (Schmidel, 1793) and in Provence and Montmorency in France (Candolle and Lamarck, 1805), it was also described in eastern North America (Muhlenberg, 1813), and noted by Robert Brown in Australia [(Brown and Bauer, 1814); Brown was botanist on the *Investigator* captained by Flinders and which circumnavigated Australia and he was the discoverer of both the nucleus and ‘Brownian’ motion], described by Joseph Dalton Hooker in the North Island of New Zealand (Hooker, 1855), collected as early as 1840 in Omgeni, Durban, South Africa (Drège, 1843) and as early as 1839 at Porto Alegre in southern Brazil (Montagne, 1839), and identified in Japan in the early 1850’s (Perry et al., 1856). These early observations suggest its presence in these locales likely predated most human mediated dispersal. However, it was noted as early as the mid-19^th^ century that *Ricciocarpos* was a suitable aquarium plant (Collier and Hooper, 1866) and this may have contributed to its dissemination in some local contexts. In regional floras, *Ricciocarpos* has been reported to be widely dispersed across the Americas, Eurasia, Africa, Australia and New Zealand [e.g. (Kashyap, 1929; Hassel de Menendez, 1962; Campbell, 1975; Scott, 1985; Piippo, 1990; Schuster, 1992; Fischer, 1995; Perold, 1995; Bischler-Causse et al., 2005; Frey et al., 2006; Lee and Gradstein, 2021; Acuña-Castillo et al., 2023)], with recent iNaturalist observations consistent with the published distribution (**Figure 1B**).

*Ricciocarpos* is thought to have diverged from its nearest extant relatives (the genera *Oxymitra* and *Riccia*) in the mid-Cretaceous (Villarreal et al., 2016), postdating the breakup of Pangea and indicating its present distribution has involved trans-oceanic dispersal. The evolution of both an aquatic lifestyle and monoicy likely evolved after the divergence of *Ricciocarpos* from *Oxymitra* and *Riccia*, and evolution of the two characters could be linked. The obvious vector for dispersal of aquatic plants over trans-oceanic distances, and shorter ones as well, is migratory waterbirds (Buch, 1954). For example, when *Ricciocarpos* was noted to be newly present at Lake Gjølsjø in eastern Norway, it was suspected that it was due to transport of plants from Swedish wetlands where *Ricciocarpos* is common, with the swan (*Cygnus olor*) the likely culprit (Skulberg, 1978). While it has not been demonstrated directly for *Ricciocarpos*, ectozoochory, including transequatorial dispersal of bryophyte diaspores in bird plumage has been documented (Lewis et al., 2014a). Endozoochory of moss spores or plant fragments *via* a number of bird species [e.g. upland goose (*Chloephaga picta*), white-bellied seedsnipe (*Attagis malouinus*), mallard (*Anas platyrhnchos*), skua (*Stercororius* sp.)] and even a flying fox (*Pteropus conspicillatus*), has been shown to be feasible (Parsons et al., 2007; Wilkinson et al., 2017; Lázaro et al., 2021; Maggio et al., 2022), and *Ricciocarpos* has been noted to be present in the faeces of mallards (Hartman, 1985). Thus, long distance dispersal *via* bird vectors (Viana et al., 2016) is a plausible mechanism to explain bryophyte species with disjunct, sometimes bipolar, geographic distributions (Schuster, 1983; Piñeiro et al., 2012; Lewis et al., 2014b). As *Ricciocarpos* is monoicous, only a single spore or thallus fragment is sufficient for dispersal to a new habitat, with evolution of monoicy an adaptation to its aquatic habit. Given the worldwide distribution of *Ricciocarpos* and its monoicous nature, it would be of interest to investigate the regional and global phylogenetic structure of the species and whether local adaptation can precede faster than dispersal.

In nature, the habitat of *Ricciocarpos* is limited to stagnant ponds and billabongs and their margins. Growth is most conspicuous during the summer months when plants may cover a significant fraction of the surface area. As temporary pools dry, the plants may become stranded on the margins, shifting to the terrestrial form with rhizoids anchoring the plants to the soil. If the waterholes refill while these stranded plants are still alive, pieces of plants originally stuck to the substrate will break free, possibly due to further growth being of the aquatic form, and the free thallus fragments can float to the pond surface once again. A similar scenario seems to occur in ponds that do not dry out, but freeze over. In this case plants growing in late autumn often sink to the bottom of the pond and over-winter there. With the coming of spring, as photosynthesis resumes, the plants then float back to the pond surface (Pickett, 1925). After some vegetative growth, first antheridia and then archegonia are produced in the late spring, with sporophytes maturing in the early summer (Garber, 1904; Pickett, 1925). If the population is undergoing the sexual life cycle, the spores can also act as over-wintering or desiccation tolerant propagules. Spores can apparently remain in the ‘seed’ bank for several years, with germination of *Ricciocarpos* observed following a ten year drought at Lake Ita, an ephemeral floodplain lake of the Lachlan River in the outback of southwestern New South Wales (Kelleway et al., 2021). Most reports suggest that *Ricciocarpos* primarily progresses through the sexual life cycle only in the aquatic form (Garber, 1904; Pickett, 1925; Maeda et al., 2016), but others have reported sexual reproduction in the terrestrial form (Lewis, 1906). One possible explanation is that sexual organs develop on the aquatic form, with sporophytes sometimes maturing after plants become stranded on the bank.

*Ricciocarpos* is often found in conjunction with a number of aquatic plants (often invasive weeds) including the angiosperms *Lemna*, *Wolffia*, *Spirodela* (i.e. the duckweeds), and *Utricularia* (the bladderworts), the fern *Azolla*, and in the northern hemisphere, another aquatic liverwort, *Riccia fluitans* (Scott, 1985; Schuster, 1992; Aoki et al., 2017). The specific community accompanying *Ricciocarpos* has been termed ‘Ricciocarpetum natantis’ (Scoppola et al., 1988), but to co-occurrence of different combination of free-floating, or pleustonic, plants appears to be random (Wolek, 1997; Wolek and Walanus, 2000). Competition among pleustonic plants is driven at least in part by nutrient supply (Peeters et al., 2016), with some evidence that increased eutrophication of Finnish lakes has facilitated the establishment and spread of *Ricciocarpos* where it had not been reported until after the 1930’s (Toivonen, 1985).

A number of studies have registered the effects of water contaminants on *Ricciocarpos* growth. Pollution of waterways by factories producing the auxin analogues 2,4-D and dikamba led to the loss of severe reduction in local populations of both *Ricciocarpos* and *R. fluitans* in Lower Silesia, Poland (Kolon and Sarosiek, 1995). In transplantation experiments from fresh to water polluted with detergents or high nitrogen concentrations, *Ricciocarpos* was less tolerant than vascular aquatic plants and succumbed in the polluted water (Agami et al., 1976). However, that *Ricciocarpos* might tolerate moderate levels of certain water contaminants was suggested by its growth in a coal strip mine impoundment in Illinois (Chimney, 1984). Growth of *Ricciocarpos* in different concentrations of heavy metals (zinc, copper, lead, cobalt, chromium, nickel and vanadium) induced specific phenotypic responses suggesting the plant might be used as a bioindicator of chemical water pollution (Sarosiek et al., 1987a; Sarosiek et al., 1987b), as did subsequent experiments with cadmium (Oh and Koh, 2013). All these heavy metals, along with manganese and aluminium (Gimenes et al., 2020), affect growth when at higher concentrations. An open question is whether *Ricciocarpos* can accumulate any to provide a tool for phytoremediation. In a similar vein, experiments demonstrate that *Ricciocarpos* exhibits high ciprofloxacin (an antibiotic) tolerance, with a capacity for uptake and accumulation despite ciprofloxacin negatively affecting photosynthetic capacity (Gomes et al., 2018). *Ricciocarpos*, and aquatic plants in general, likely evolved mechanisms to cope with water contaminants, either internally, or alternatively involving active secretion of chemicals to modify their immediate environment, both chemically and altering the microbiome composition. *Ricciocarpos* may be a comparable model for such studies along with duckweeds (Fourounjian et al., 2020) and *Azolla* (Li et al., 2018).

## Culture of Ricciocarpos

*Ricciocarpos* is amenable to growth in axenic culture under a variety of growth conditions (Woodfin, 1976; Lorenzen et al., 1981; Kunz and Becker, 1995). A culture of *Ricciocarpos natans* was obtained from Dr. Christine Cargill, curator of cryptogam collections at the Australian National Botanic Gardens in Canberra. This culture was originally isolated from Jerrabomberra Creek, near a bridge over the creek in Jerrabomberra Wetlands in the Australian Capital Territory (35° 18’ S, 149° 9’ E). We established axenic cultures with conditions adapted to our growth rooms. The aquatic form of *Ricciocarpos* grows on the surface of stagnant water bodies, such as ponds and billabongs, conditions which are not necessarily nutrient rich. Thus, *Ricciocarpos* was grown with varying concentrations of Gamborg B-5 media, pH 6.0, (Gamborg et al., 1968) to identify a ‘wild-like’ morphology of aquatic form of *Ricciocarpos*, based on previous descriptions of the species growing in nature. Plants were grown for 4 weeks and plant morphology, as well as the quantity of growth produced, with each varying nutrient concentration was compared. A peak of growth, measured by surface area, was observed when plants were grown in 1/8 B-5 media (**Figure 3A**). Increasing light intensity from 45 µmol. m^-2^s^-1^ to 80 µmol. m^-2^s^-1^ was sufficient to induce the production of pigmented scales on the ventral surface (**Figure 3B**); this range is similar to light intensities used in some previous *in vitro* culture conditions (Lorenzen et al., 1981; Kunz and Becker, 1995).

## Genome

We recently reported an assembly of the *Ricciocarpos natans* genome based on approximately 200x coverage of Illumina sequencing followed by scaffold assembly using Hi-C [available at https://genomevolution.org/; (Singh et al., 2023)]. The current version (v1.0) consists of 38 large scaffolds covering 185.50 Mb genome assembly. Structural annotation of *Ricciocarpos* genome revealed 18,813 protein coding genes in *Ricciocarpos*, which is similar to gene number with the reference species *Marchantia polymorpha ruderalis*, hereafter *Marchantia*, with 19,473 genes (Bowman et al., 2017; Montgomery et al., 2020). *Marchantia* has 23,399 proteins and *Ricciocarpos* has 21,958 proteins suggesting that *Ricciocarpos* has 3,145 additional isomers. Blast searches to identify how many *Ricciocarpos* proteins have orthologs in *Marchantia* were performed using Blast-P with the *Ricciocarpos* protein set against the *Marchantia* protein set with filter parameters of percentage identity >30%, bitscore of >50 and e-value <=1e-05. This revealed that out of total number of 21,958 *Ricciocarpos* proteins orthologs were identified for 13,910 proteins

The accession from which the genome presented is derived was isolated from Jerrybomberra Creek, ACT, Australia, while the1kp transcriptome data was derived from mRNA isolated from a plant from near Butner, NC, USA. Their geographically distinct origins afforded the opportunity to examine sequence divergence between the two accessions. Surprisingly, it was noted that the two accessions differ on average by approximately 4% in the coding regions analysed, which is more than is typically observed for individual comparisons within eukaryotic species (<1%), and is closer to upper values of combined divergence for populations (Leffler et al., 2012) and to that (5%) observed for bacterial ‘species’ (Jain et al., 2018). The diversification of subspecies of the *Marchantia polymorpha* complex are thought to date to the late Miocene, 5 Ma (highest posterior density 2-11 Ma) (Villarreal et al., 2016). The single nucleotide polymorphism frequency between *Marchantia polymorpha* subspecies is approximately 1.0-1.2% (Linde et al., 2020), while that between the Australian and North American *Ricciocarpos* isolates is approximately 4.1%. In the absence of fossil calibration, the nucleotide differences suggest divergence of the two *Ricciocarpos* populations perhaps in the early Miocene, 15-20 Ma.

We examined the phylogenetic relationships between *Ricciocarpos natans* accessions for which DNA sequence was available on Genbank, and found that sequences were distributed into at least two distinct clades (**Figure 1C**). One clade contained sequences from five accessions collected in Australia and Asia, including the Jerrybomberra Creek accession, while a second distinct clade was composed of sequences representing five accessions collected in North America. Ironically, a paucity of available DNA sequence from accessions collected in Europe, where *Ricciocarpos* was first described, prevented definitive placement of European accessions relative to the distinct two clades, with a single sequence with a long branch representing this continent. Given the sequence divergence between the Australasian and North American accessions, we suggest that these two clades might represent two distinct reproductively isolated *Ricciocarpos* populations and could be considered two separate species. Further work is required to ascertain whether there exist morphological or anatomical characters that define the two clades. Whether the European accessions might represent a third entity must await more sequence data from such accessions. Regardless of the phylogenetic position of the European accessions, it is of note that the divergence between the American and Australasian accessions could be related to distinct migratory bird flyways (Boere and Stroud, 2006). In this regard, it will be of interest whether genetic characterization of *Ricciocarpos* accessions from other geographic locations, such as South America, Africa, Central Asia and New Zealand also correlate with migratory patterns of birds.

## The aquatic to terrestrial morphological transition in *Ricciocarpos*

*Ricciocarpos* is secondarily adapted to aquatic life and is found worldwide in stagnant water, e.g. ponds and billabongs. However, as water levels drop seasonally, *Ricciocarpos* plants may become stranded on the terrestrial margins. *Ricciocarpos* exhibits strong morphological differences based on the habitat in which it is growing, with a plant of the same genotype being able to transition from a free-floating aquatic form into a terrestrial form (Garber, 1904; Pickett, 1925). When growing in culture, the addition of abscisic acid (ABA) to the media of aquatically growing *Ricciocarpos* is sufficient to induce the transformation in growth habit from the aquatic to that of the terrestrial form (Hartung et al., 1994). This observation parallels similar experiments on another secondarily aquatic liverwort, *Riccia fluitans*, that usually grows submerged in water rather than floating on the surface. When *Riccia fluitans* is transferred from an aquatic medium to one exposed to the air, a transition from an aquatic growth form to a terrestrial form and during this transition process the concentration of ABA is increased 10-30 fold (Hellwege et al., 1992). Furthermore, treatment of *Riccia fluitans* with ABA can induce such characteristics even when the thallus is submerged in water (Hellwege et al., 1992), with concomitant changes in gene expression, including genes encoding Late Embryogenesis Abundant (LEA) proteins (Hellwege et al., 1996). The transition, which takes place over a couple weeks, includes changes to cell division patterns at the shoot apex such that the terrestrial form has larger air chambers and some air pores, in addition to the development of rhizoids (Althoff et al., 2022).

**Figure 4:**
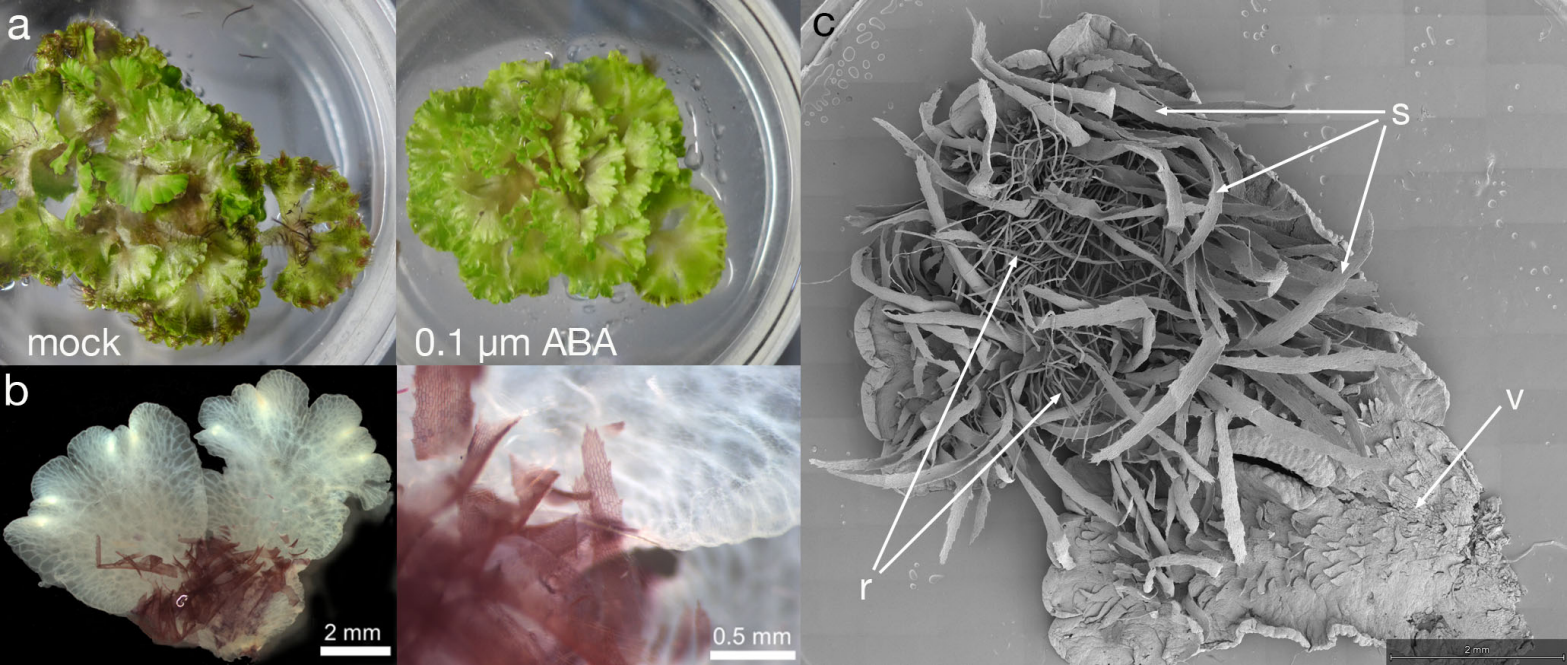
ABA induced formation of the terrestrial form. **(A)** When grown aquatically in ⅛ Gamborg B-5 media, plants produce copious ventral long scales, with their purple colour visible in the mocked plants (left); some of the thalli are flipped over to show the ventral scales. ABA treatment of leads to growth of the terrestrial form (right), lacking purple scales and. The diameter of petri plate is 9 cm. **(B)** The aquatic form of *Ricciocarpos* exhibits purple scales. The purple pigment of scales is cell wall bound, perhaps providing structural support. The image shows a thallus fixed in Formaldehyde Alcohol Acetic Acid - FAA and cleared in 100% Ethanol. **(C)** Both scales and rhizoids are observed in *Ricciocarpos* following a shift to ABA containing media. This plant was initially producing ventral scales, but began to produce rhizoids after ABA treatment; if treatment continues, eventually the scales will fall off and the plant will only have rhizoids; ventral thallus; s, scale; r, rhizoid.

To further understand the aquatic to terrestrial transition in *Ricciocarpos*, we repeated earlier observations. In our growth conditions the morphological differences between aquatic and terrestrial forms of *Ricciocarpos* were successfully induced by addition of 0.1µM ABA (**Figure 4A**). The aquatic form is characterized by the development of long ventral scales that act as keels to keep the plants stable on the water surface (**Figures 4B, C**). The scales are usually heavily pigmented with riccionidin (**Figure 4B**), which is an auronidin localized to the cell wall and whose synthesis is biochemically related to that of anthocyanins that are common in other land plants (Kunz et al., 1994; Albert et al., 2018; Berland et al., 2019). In the aquatic form, the development of rhizoids is suppressed. Conversely, in the terrestrial form, the development of rhizoids is promoted, while that of scales is repressed. In addition, separation of the thallus *via* (presumably) programmed cell death, following dichotomous branching is a form of vegetative reproduction that allows dispersal of the plants across the water surface. Separation is suppressed in the terrestrial form such that plants form a rosette. When ABA is added to aquatically growing plants, a transition from production of scales to the production of rhizoids is observed and separation of thalli is suppressed (**Figures 4A, C**).

**Figure 5:**
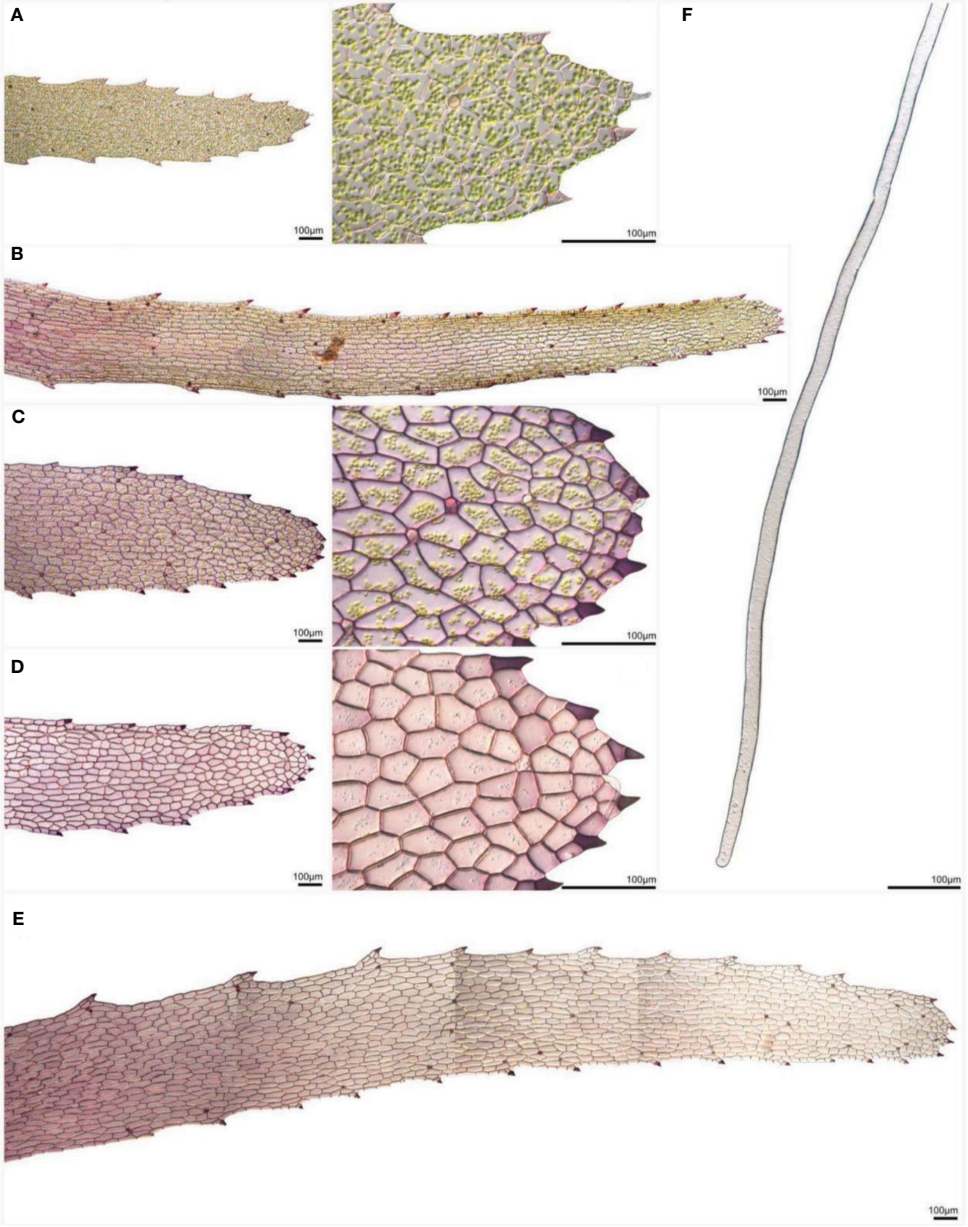
Scales and rhizoids of *Ricciocarpos.* **(A)** An immature scale in which little riccionidin is visible. (a’) Magnification of **(A)** showing conspicuous chloroplasts. **(B)** In slightly older scales, the reddish-purple coloured riccionidin pigment initially appears at the proximal end of the scale and progresses towards the distal tip. **(C, D)** As scales differentiate, an increase in riccionidin pigmentation is distinctly visible. (c’ and d’) Magnifications of **(C, D)**, respectively, showing the loss of chloroplasts as scales mature. **(E)** Mature scales retain a gradient of pigmentation. **(F)** *Ricciocarpos* rhizoids are similar to the smooth rhizoids of *Marchantia* (Shimamura, 2016; Bowman et al., 2022).

In the aquatic form, the scales that develop from the ventral epidermis are deeply pigmented with riccionidin at maturity. However, immature scales in which cell division is still occurring lack riccionidin pigmentation, but do contain conspicuous chloroplasts (**Figure 5A**). As scales differentiate, riccionidin accumulation first appears proximally and then gradually extends to the distal tip (**Figure 5B**). As scales mature riccionidin accumulation continues and chloroplasts are no longer conspicuous (**Figures 5C-E**). Pigmentation is particularly intense in the marginal cells with protuberances giving the scale a fringed appearance (**Figures 5C-E**). As the riccionidin accumulation occurs as a polymerized derivative embedded in the cell wall (Kunz et al., 1994; Berland et al., 2019), it may act to stiffen the scales to aid their function as keels. The production of the cell wall pigment may also preclude subsequence cell division (Albert et al., 2018). In the terrestrial form the scales remain small and are restricted to the region of the apical meristem (Kronestedt, 1982a). The rhizoids that develop in the terrestrial form following ABA treatment are smooth rhizoids (**Figure 5F**)

**Figure 6:**
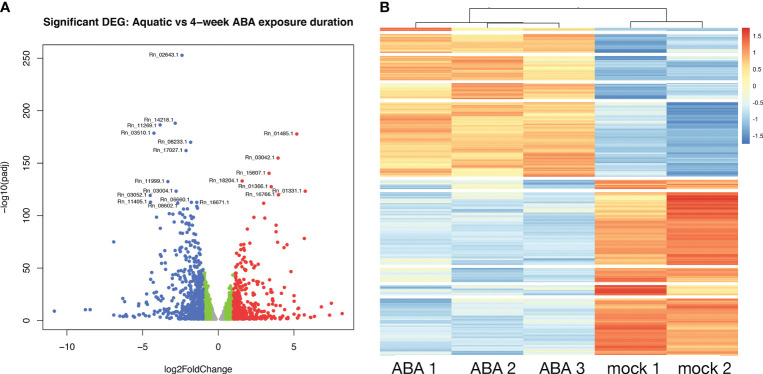
Differential gene expression between aquatic and terrestrial forms. **(A)** Differentially expressed genes in Aquatic vs 4-week ABA treated samples: adjusted p-values of <0.05 are plotted in the above graph with log2Foldchange between -0.5 to 0.5 represented in grey, genes with log2Foldchange in range -0.5 to -1 and 0.5 to 1 shown in green. The down-regulated and up-regulated genes with log2Foldchange greater than 1 or smaller than -1 are shown in red and blue color, respectively. **(B)** Heatmap showing differential gene expression in ABA treated *Ricciocarpos* in contrast to the control sample with no ABA treatment. Orange color signifies up regulated genes and blue color signifies down-regulated genes, scaled to normalized read counts.

To explore the role of ABA on gene expression in the *Ricciocarpos* thallus during the transition from an aquatic form to a terrestrial form, a differential expression analysis comparing plants growing aquatically to those grown for four weeks in the presence of ABA [0.1µM] was performed; given the time point the differentially expressed genes (DEG) will represent steady state levels following long-term ABA exposure. DEG were identified considering all data and observing the scaling at the gene level to identify differential expression (up or down). This analysis revealed that out of 15,440 genes with non-zero total read count with adjusted p-value < 0.05, a total of 2237 (15% of genes) are up-regulated (log2Foldchange > 0) and 2798 (18% of genes) are down-regulated (log2Foldchange < 0) (**Figure 6A**). A heatmap of the DEG facilitated hierarchical clustering to identify gene clusters displaying similar expression patterns amongst samples (**Figure 6B**). The heatmap was plotted with all significant genes with adjusted value < 0.05. The genes differentially expressed are grouped in 10 clusters.

*Response of ABA related Genes:* To determine changes in ABA-related genes, transcriptomes were analysed to identify expression of each known ortholog of *Marchantia* ABA biosynthesis and response genes (Bowman et al., 2017). DEG analysis of ABA-related genes revealed Rn_08915.1, an ortholog of *Marchantia* MpNCED (9-cis-epoxycarotenoid dioxygenase1; Mp2g07800) and of NCED1 (At3g63520) of Arabidopsis (Bowman et al., 2017), is up-regulated upon ABA treatment (**Table 1**). In contrast, the PYRABACTIN RESISTANCE1-like (PYR1-like) ABA receptor, an ortholog of MpPYL1 and the fourteen PYR-related genes of Arabidopsis (Bowman et al., 2017; Jahan et al., 2019) was not found to be differentially expressed when comparing aquatic and ABA treated samples.

**Table 1:** ABA genes differentially expressed with ABA treated plants in comparison to aquatic *R. natans*.

Marchantia polymorpha Gene ID	Rn Gene ID	4 week	Gene Symbol	GO/PANTHER
Mp2g07800.1	Rn_08915.1	UP	Mp*NCED*: 9-cis-epoxycarotenoid dioxigenase	GO:0016702: oxidoreductase activity, acting on single donors with incorporation of molecular oxygen, incorporation of two atoms of oxygen
Mp1g24460.1	Rn_14445.1	X	Mp*SNRK2A*: SNF1-related protein kinase2	GO:0004672: protein kinase activity
Mp8g06370.1	Rn_10037.2	UP	Mp*AO*: abscisic aldehyde oxidase	GO:0016491: oxidoreductase activity
Mp2g25940.1	Rn_07898.1	DOWN	MpCYP707A: ABA 8’-hydroxylase	GO:0016705: oxidoreductase activity, acting on paired donors, with incorporation or reduction of molecular oxygen
Mp2g00670.1	Rn_08366.1	DOWN	MpABA1:	GO:0009688: abscisic acid biosynthetic process
			zeaxanthin epoxidase	
Mp8g17460.1	Rn_17318.1	X	MpPYL1: PYR1-like abscisic acid receptor	PTHR31213:SF119 : ABSCISIC ACID RECEPTOR PYL4

X denotes not differentially expressed.

*ABA control expression of LEA-like genes:* ABA reduces growth and enhances desiccation tolerance by increasing accumulation of intracellular sugars and various proteins such as those encoded by LEA-like genes (Akter et al., 2014; Hernández-Sánchez et al., 2022). Expression patterns of *Ricciocarpos* LEA genes are presented in **Table 2**. As expected, there is accumulation of several LEA genes upon ABA treatment, with Rn_05573.1 (LEA_1), Rn_18585.1 (LEA_1), and Rn_15939.1 (LEA_4) up-regulated in response to exogenous ABA application and Rn_16212.1 (LEA_2) and Rn_12186.1 (LEA_2) downregulated; LEA families as previously defined (Artur et al., 2018). In addition, several members of the NHL (*NDR1*, nonrace specific disease resistance 1; *HIN1*, hairpin-induced 1) gene family previously associated with disease resistance and ABA response (Bao et al., 2016) were differentially regulated, with Rn_11497.1, Rn_08770.1, and Rn_09383.1 downregulated in response to exogenous ABA.

**Table 2:** LEA genes are differentially expressed with ABA treated plants in comparison to aquatic *R. natans*.

Marchantia polymorpha Gene ID	Rn Gene ID	4 week	Gene Symbol	LEA group	GO/PANTHER
Mp1g17670.1		DOWN	No gene symbols are registered for this gene	LEA_2	PTHR31852:SF141:LATE EMBRYOGENESIS ABUNDANT PROTEIN, GROUP 2; PANTHER:PTHR31852:LATE EMBRYOGENESIS ABUNDANT (LEA) HYDROXYPROLINE-RICH GLYCOPROTEIN FAMILY
	Rn_16212.1				
Mp3g04820.1	Rn_05573.1	UP	MpLEA-like12:Late-embryogenesis abundant protein	LEA_1	GO:0009793:embryo development ending in seed dormancy;

Mp7g19340.1	Rn_11497.1	DOWN	MpLEA-like58:	NHL	PANTHER:PTHR31234:LATE EMBRYOGENESIS ABUNDANT (LEA) HYDROXYPROLINE-RICH GLYCOPROTEIN FAMILY
			Late-embryogenesis abundant protein		
Mp3g23740.1	Rn_18585.1	UP	MpLEA-like16:Late-embryogenesis abundant protein	LEA_4	GO:0009793:embryo development ending in seed dormancy

Mp1g01170.1	Rn_15939.1		MpLEA-like2:Late-embryogenesis abundant protein	LEA_4	**PTHR47652:SF3**, LATE EMBRYOGENESIS ABUNDANT PROTEIN (LEA) FAMILY PROTEIN
		UP			
Mp2g12370.1	Rn_08770.1	DOWN	MpLEA-like8:Late-embryogenesis abundant protein	NHL	PANTHER:PTHR31234:LATE EMBRYOGENESIS ABUNDANT (LEA) HYDROXYPROLINE-RICH GLYCOPROTEIN FAMILY
Mp8g07630.1	Rn_09383.1	DOWN	MpLEA-like59:Late-embryogenesis abundant protein	NHL	PANTHER:PTHR31234:LATE EMBRYOGENESIS ABUNDANT (LEA) HYDROXYPROLINE-RICH GLYCOPROTEIN FAMILY

Mp7g12850.1	Rn_12186.1	DOWN	MpLEA-like56:Late-embryogenesis abundant protein	LEA_2	PTHR31852:SF180:PROTEIN, PUTATIVE-RELATED

*Expression of the rhizoid gene*, RSL1: The *Marchantia* gene Mp*RSL1* (Mp3g17930.1) is required for rhizoid initiation and is an ortholog of Arabidopsis *ROOT HAIR DEFECTIVE6* that acts in root hair development (Proust et al., 2016). The orthologous *Ricciocarpos* gene is Rn_00421, and consistent with the observed morphology of ABA treated *Ricciocarpos* plants, this gene is up-regulated upon ABA treatment. An analysis examining gene expression at shorter intervals during the aquatic to terrestrial transition should identify additional gene regulatory networks associated with the developmental changes.

Several limnetic or riparian plants exhibit phenotypic plasticity wherein morphology is dependent upon environmental conditions, e.g. whether growing submerged or floating on the water versus aerial growth or on a terrestrial substrate. In many cases the shift from an aquatic morphology to a terrestrial morphology is correlated or mediated with increased endogenous ABA levels. For example, in two liverworts that evolved a secondarily aquatic habit independently, *Riccia fluitans* and *Ricciocarpos natans*, the terrestrial form can be induced by ABA (Hellwege et al., 1992; Hartung et al., 1994; Hellwege et al., 1996; Althoff et al., 2022). In each of these species, the terrestrial form develops rhizoids, while the aquatic form does not, but other morphological changes appear to be species specific, such as the loss of scale production and loss of thallus separation appear to be specific to *Ricciocarpos*. Similar aquatic-terrestrial transitions are also observed in vascular plants in the form of heterophylly, wherein leaf morphology is polymorphic depending upon environmental conditions (Nakayama et al., 2017; Li et al., 2019). Such heterophylly has evolved multiple times independently within angiosperms, and while in many instances ABA in implicated, its involvement is not universal. For example, abscisic acid induces formation of floating leaves in the heterophyllous aquatic angiosperm *Potamogeton nodosus* (Anderson, 1978) and abscisic acid induces land form characteristics in the fern *Marsilea quadrifolia* (Liu, 1984). In contrast, aquatic-terrestrial heterophylly in *Rorippa aquatica* another phytohormone, gibberellic acid, regulates leaf morphology in conjunction with KNOX1 genes known to influence leaf complexity (Nakayama et al., 2014) and in *Ranunculus trichophyllus* ABA works in conjunction with a third phytohormome, ethylene, to regulate heterophylly (Kim et al., 2018). Ethylene has also been implicated in heterophylly in other angiosperm species (Nakayama et al., 2017; Li et al., 2019). Both ethylene and ABA are implicated in adaptations to water stress, too much for the former and too little for the latter (Jackson, 2008; Sakata et al., 2013). Thus, these phytohormone signalling pathways are likely already active in the tissues that will develop heterophylly or other developmental modifications in aquatic plants, and can be integrated into gene regulatory networks controlling morphology as well as physiology.

## Summary

While we developed *Ricciocarpos natans* as a ‘model’ system for examining genomic evolution during the transition for dioicy to monoicy (Singh et al., 2023), once some tools are developed for a species, other biological questions come calling. In today’s age, only a few prerequisites are required for establishing a model organism — e.g. ease of growth in culture, genomic resources, ability to introduce genome editing technologies. For *Ricciocarpos*, the former two have been established and it is likely that transformation using protocols for the related species, *Riccia fluitans* (Althoff and Zachgo, 2020), can be readily adapted to *Ricciocarpos*. Thus, *Ricciocarpos* is primed to be utilized to answer a spectrum of biological questions only limited by the imagination, and of course funding.

## Data availability statement

The data presented in the study are deposited at https://genomevolution.org/coge/, accession code: ‘Ricciocarpos’.

## Author contributions

SS: Conceptualization, Formal Analysis, Investigation, Methodology, Writing – original draft. JB: Conceptualization, Formal Analysis, Funding acquisition, Supervision, Writing – original draft, Writing – review & editing.
